# Updated resources for exploring experimentally-determined PDB structures and Computed Structure Models at the RCSB Protein Data Bank

**DOI:** 10.1093/nar/gkae1091

**Published:** 2024-11-28

**Authors:** Stephen K Burley, Rusham Bhatt, Charmi Bhikadiya, Chunxiao Bi, Alison Biester, Pratyoy Biswas, Sebastian Bittrich, Santiago Blaumann, Ronald Brown, Henry Chao, Vivek Reddy Chithari, Paul A Craig, Gregg V Crichlow, Jose M Duarte, Shuchismita Dutta, Zukang Feng, Justin W Flatt, Sutapa Ghosh, David S Goodsell, Rachel Kramer Green, Vladimir Guranovic, Jeremy Henry, Brian P Hudson, Michael Joy, Jason T Kaelber, Igor Khokhriakov, Jhih-Siang Lai, Catherine L Lawson, Yuhe Liang, Douglas Myers-Turnbull, Ezra Peisach, Irina Persikova, Dennis W Piehl, Aditya Pingale, Yana Rose, Jared Sagendorf, Andrej Sali, Joan Segura, Monica Sekharan, Chenghua Shao, James Smith, Michael Trumbull, Brinda Vallat, Maria Voigt, Ben Webb, Shamara Whetstone, Amy Wu-Wu, Tongji Xing, Jasmine Y Young, Arthur Zalevsky, Christine Zardecki

**Affiliations:** Research Collaboratory for Structural Bioinformatics Protein Data Bank, Rutgers, The State University of New Jersey, Piscataway, NJ 08854, USA; Institute for Quantitative Biomedicine, Rutgers, The State University of New Jersey, Piscataway, NJ 08854, USA; Rutgers Cancer Institute, New Brunswick, NJ 08901, USA; Department of Chemistry and Chemical Biology, Rutgers, The State University of New Jersey, Piscataway, NJ 08854, USA; Research Collaboratory for Structural Bioinformatics Protein Data Bank, San Diego Supercomputer Center, University of California, La Jolla, CA 92093, USA; Rutgers Artificial Intelligence and Data Science (RAD) Collaboratory, Rutgers, The State University of New Jersey, Piscataway, NJ 08854, USA; Research Collaboratory for Structural Bioinformatics Protein Data Bank, Rutgers, The State University of New Jersey, Piscataway, NJ 08854, USA; Institute for Quantitative Biomedicine, Rutgers, The State University of New Jersey, Piscataway, NJ 08854, USA; Research Collaboratory for Structural Bioinformatics Protein Data Bank, San Diego Supercomputer Center, University of California, La Jolla, CA 92093, USA; Research Collaboratory for Structural Bioinformatics Protein Data Bank, San Diego Supercomputer Center, University of California, La Jolla, CA 92093, USA; Research Collaboratory for Structural Bioinformatics Protein Data Bank, Rutgers, The State University of New Jersey, Piscataway, NJ 08854, USA; Institute for Quantitative Biomedicine, Rutgers, The State University of New Jersey, Piscataway, NJ 08854, USA; Research Collaboratory for Structural Bioinformatics Protein Data Bank, Rutgers, The State University of New Jersey, Piscataway, NJ 08854, USA; Institute for Quantitative Biomedicine, Rutgers, The State University of New Jersey, Piscataway, NJ 08854, USA; Research Collaboratory for Structural Bioinformatics Protein Data Bank, San Diego Supercomputer Center, University of California, La Jolla, CA 92093, USA; Research Collaboratory for Structural Bioinformatics Protein Data Bank, Rutgers, The State University of New Jersey, Piscataway, NJ 08854, USA; Institute for Quantitative Biomedicine, Rutgers, The State University of New Jersey, Piscataway, NJ 08854, USA; Research Collaboratory for Structural Bioinformatics Protein Data Bank, Rutgers, The State University of New Jersey, Piscataway, NJ 08854, USA; Institute for Quantitative Biomedicine, Rutgers, The State University of New Jersey, Piscataway, NJ 08854, USA; Research Collaboratory for Structural Bioinformatics Protein Data Bank, Rutgers, The State University of New Jersey, Piscataway, NJ 08854, USA; Institute for Quantitative Biomedicine, Rutgers, The State University of New Jersey, Piscataway, NJ 08854, USA; Research Collaboratory for Structural Bioinformatics Protein Data Bank, Rutgers, The State University of New Jersey, Piscataway, NJ 08854, USA; Institute for Quantitative Biomedicine, Rutgers, The State University of New Jersey, Piscataway, NJ 08854, USA; School of Chemistry and Materials Science, Rochester Institute of Technology, Rochester, NY 14623, USA; Research Collaboratory for Structural Bioinformatics Protein Data Bank, Rutgers, The State University of New Jersey, Piscataway, NJ 08854, USA; Institute for Quantitative Biomedicine, Rutgers, The State University of New Jersey, Piscataway, NJ 08854, USA; Research Collaboratory for Structural Bioinformatics Protein Data Bank, San Diego Supercomputer Center, University of California, La Jolla, CA 92093, USA; Research Collaboratory for Structural Bioinformatics Protein Data Bank, Rutgers, The State University of New Jersey, Piscataway, NJ 08854, USA; Institute for Quantitative Biomedicine, Rutgers, The State University of New Jersey, Piscataway, NJ 08854, USA; Rutgers Cancer Institute, New Brunswick, NJ 08901, USA; Research Collaboratory for Structural Bioinformatics Protein Data Bank, Rutgers, The State University of New Jersey, Piscataway, NJ 08854, USA; Institute for Quantitative Biomedicine, Rutgers, The State University of New Jersey, Piscataway, NJ 08854, USA; Research Collaboratory for Structural Bioinformatics Protein Data Bank, Rutgers, The State University of New Jersey, Piscataway, NJ 08854, USA; Institute for Quantitative Biomedicine, Rutgers, The State University of New Jersey, Piscataway, NJ 08854, USA; Research Collaboratory for Structural Bioinformatics Protein Data Bank, Rutgers, The State University of New Jersey, Piscataway, NJ 08854, USA; Institute for Quantitative Biomedicine, Rutgers, The State University of New Jersey, Piscataway, NJ 08854, USA; Research Collaboratory for Structural Bioinformatics Protein Data Bank, Rutgers, The State University of New Jersey, Piscataway, NJ 08854, USA; Institute for Quantitative Biomedicine, Rutgers, The State University of New Jersey, Piscataway, NJ 08854, USA; Rutgers Cancer Institute, New Brunswick, NJ 08901, USA; Department of Integrative Structural and Computational Biology, The Scripps Research Institute, La Jolla, CA 92037, USA; Research Collaboratory for Structural Bioinformatics Protein Data Bank, Rutgers, The State University of New Jersey, Piscataway, NJ 08854, USA; Research Collaboratory for Structural Bioinformatics Protein Data Bank, Rutgers, The State University of New Jersey, Piscataway, NJ 08854, USA; Institute for Quantitative Biomedicine, Rutgers, The State University of New Jersey, Piscataway, NJ 08854, USA; Research Collaboratory for Structural Bioinformatics Protein Data Bank, San Diego Supercomputer Center, University of California, La Jolla, CA 92093, USA; Research Collaboratory for Structural Bioinformatics Protein Data Bank, Rutgers, The State University of New Jersey, Piscataway, NJ 08854, USA; Institute for Quantitative Biomedicine, Rutgers, The State University of New Jersey, Piscataway, NJ 08854, USA; Research Collaboratory for Structural Bioinformatics Protein Data Bank, Rutgers, The State University of New Jersey, Piscataway, NJ 08854, USA; Institute for Quantitative Biomedicine, Rutgers, The State University of New Jersey, Piscataway, NJ 08854, USA; Research Collaboratory for Structural Bioinformatics Protein Data Bank, Rutgers, The State University of New Jersey, Piscataway, NJ 08854, USA; Institute for Quantitative Biomedicine, Rutgers, The State University of New Jersey, Piscataway, NJ 08854, USA; Research Collaboratory for Structural Bioinformatics Protein Data Bank, San Diego Supercomputer Center, University of California, La Jolla, CA 92093, USA; Research Collaboratory for Structural Bioinformatics Protein Data Bank, San Diego Supercomputer Center, University of California, La Jolla, CA 92093, USA; Research Collaboratory for Structural Bioinformatics Protein Data Bank, Rutgers, The State University of New Jersey, Piscataway, NJ 08854, USA; Institute for Quantitative Biomedicine, Rutgers, The State University of New Jersey, Piscataway, NJ 08854, USA; Research Collaboratory for Structural Bioinformatics Protein Data Bank, Rutgers, The State University of New Jersey, Piscataway, NJ 08854, USA; Institute for Quantitative Biomedicine, Rutgers, The State University of New Jersey, Piscataway, NJ 08854, USA; Research Collaboratory for Structural Bioinformatics Protein Data Bank, San Diego Supercomputer Center, University of California, La Jolla, CA 92093, USA; Research Collaboratory for Structural Bioinformatics Protein Data Bank, Rutgers, The State University of New Jersey, Piscataway, NJ 08854, USA; Institute for Quantitative Biomedicine, Rutgers, The State University of New Jersey, Piscataway, NJ 08854, USA; Research Collaboratory for Structural Bioinformatics Protein Data Bank, Rutgers, The State University of New Jersey, Piscataway, NJ 08854, USA; Institute for Quantitative Biomedicine, Rutgers, The State University of New Jersey, Piscataway, NJ 08854, USA; Research Collaboratory for Structural Bioinformatics Protein Data Bank, Rutgers, The State University of New Jersey, Piscataway, NJ 08854, USA; Institute for Quantitative Biomedicine, Rutgers, The State University of New Jersey, Piscataway, NJ 08854, USA; Research Collaboratory for Structural Bioinformatics Protein Data Bank, Rutgers, The State University of New Jersey, Piscataway, NJ 08854, USA; Institute for Quantitative Biomedicine, Rutgers, The State University of New Jersey, Piscataway, NJ 08854, USA; Research Collaboratory for Structural Bioinformatics Protein Data Bank, San Diego Supercomputer Center, University of California, La Jolla, CA 92093, USA; Research Collaboratory for Structural Bioinformatics Protein Data Bank, Department of Bioengineering and Therapeutic Sciences, Department of Pharmaceutical Chemistry, Quantitative Biosciences Institute, University of California, San Francisco, CA 94158, USA; Research Collaboratory for Structural Bioinformatics Protein Data Bank, Department of Bioengineering and Therapeutic Sciences, Department of Pharmaceutical Chemistry, Quantitative Biosciences Institute, University of California, San Francisco, CA 94158, USA; Research Collaboratory for Structural Bioinformatics Protein Data Bank, San Diego Supercomputer Center, University of California, La Jolla, CA 92093, USA; Research Collaboratory for Structural Bioinformatics Protein Data Bank, Rutgers, The State University of New Jersey, Piscataway, NJ 08854, USA; Institute for Quantitative Biomedicine, Rutgers, The State University of New Jersey, Piscataway, NJ 08854, USA; Research Collaboratory for Structural Bioinformatics Protein Data Bank, Rutgers, The State University of New Jersey, Piscataway, NJ 08854, USA; Institute for Quantitative Biomedicine, Rutgers, The State University of New Jersey, Piscataway, NJ 08854, USA; Research Collaboratory for Structural Bioinformatics Protein Data Bank, Rutgers, The State University of New Jersey, Piscataway, NJ 08854, USA; Institute for Quantitative Biomedicine, Rutgers, The State University of New Jersey, Piscataway, NJ 08854, USA; Research Collaboratory for Structural Bioinformatics Protein Data Bank, Rutgers, The State University of New Jersey, Piscataway, NJ 08854, USA; Institute for Quantitative Biomedicine, Rutgers, The State University of New Jersey, Piscataway, NJ 08854, USA; Research Collaboratory for Structural Bioinformatics Protein Data Bank, Rutgers, The State University of New Jersey, Piscataway, NJ 08854, USA; Institute for Quantitative Biomedicine, Rutgers, The State University of New Jersey, Piscataway, NJ 08854, USA; Research Collaboratory for Structural Bioinformatics Protein Data Bank, Rutgers, The State University of New Jersey, Piscataway, NJ 08854, USA; Institute for Quantitative Biomedicine, Rutgers, The State University of New Jersey, Piscataway, NJ 08854, USA; Research Collaboratory for Structural Bioinformatics Protein Data Bank, Department of Bioengineering and Therapeutic Sciences, Department of Pharmaceutical Chemistry, Quantitative Biosciences Institute, University of California, San Francisco, CA 94158, USA; Research Collaboratory for Structural Bioinformatics Protein Data Bank, Rutgers, The State University of New Jersey, Piscataway, NJ 08854, USA; Institute for Quantitative Biomedicine, Rutgers, The State University of New Jersey, Piscataway, NJ 08854, USA; Research Collaboratory for Structural Bioinformatics Protein Data Bank, Rutgers, The State University of New Jersey, Piscataway, NJ 08854, USA; Institute for Quantitative Biomedicine, Rutgers, The State University of New Jersey, Piscataway, NJ 08854, USA; Research Collaboratory for Structural Bioinformatics Protein Data Bank, Rutgers, The State University of New Jersey, Piscataway, NJ 08854, USA; Institute for Quantitative Biomedicine, Rutgers, The State University of New Jersey, Piscataway, NJ 08854, USA; Research Collaboratory for Structural Bioinformatics Protein Data Bank, Rutgers, The State University of New Jersey, Piscataway, NJ 08854, USA; Institute for Quantitative Biomedicine, Rutgers, The State University of New Jersey, Piscataway, NJ 08854, USA; Research Collaboratory for Structural Bioinformatics Protein Data Bank, Department of Bioengineering and Therapeutic Sciences, Department of Pharmaceutical Chemistry, Quantitative Biosciences Institute, University of California, San Francisco, CA 94158, USA; Research Collaboratory for Structural Bioinformatics Protein Data Bank, Rutgers, The State University of New Jersey, Piscataway, NJ 08854, USA; Institute for Quantitative Biomedicine, Rutgers, The State University of New Jersey, Piscataway, NJ 08854, USA

## Abstract

The Research Collaboratory for Structural Bioinformatics Protein Data Bank (RCSB PDB, RCSB.org), the US Worldwide Protein Data Bank (wwPDB, wwPDB.org) data center for the global PDB archive, provides access to the PDB data *via* its RCSB.org research-focused web portal. We report substantial additions to the tools and visualization features available at RCSB.org, which now delivers more than 227000 experimentally determined atomic-level three-dimensional (3D) biostructures stored in the global PDB archive alongside more than 1 million Computed Structure Models (CSMs) of proteins (including models for human, model organisms, select human pathogens, crop plants and organisms important for addressing climate change). In addition to providing support for 3D structure motif searches with user-provided coordinates, new features highlighted herein include query results organized by redundancy-reduced Groups and summary pages that facilitate exploration of groups of similar proteins. Newly released programmatic tools are also described, as are enhanced training opportunities.

## Graphical Abstract

**Graphical Abstract:**
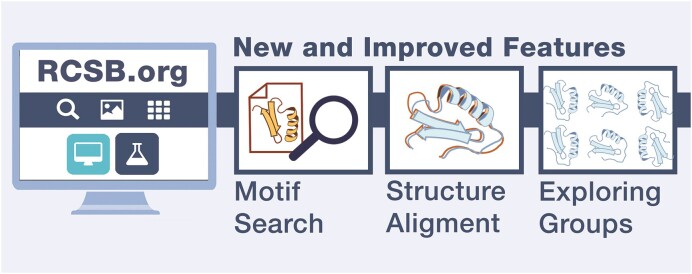

## Introduction

The Protein Data Bank (PDB) was established as the first open-access digital data resource in biology (1). It has been supported without interruption by the United States (U.S.) government since it was founded in 1971. At the time of writing in 2024, the RCSB Protein Data Bank (2) has been continuously funded by the U.S. National Science Foundation, National Institutes of Health and the U.S. Department of Energy for more than 25 years. With renewal of this joint funding for another five years in early 2024, the RCSB PDB will reach the milestone of 30 years of continuous U.S. federal funding in 2029. The RCSB PDB serves as the wwPDB data center in the U.S. for the global PDB archive of rigorously validated, expertly biocurated 3D structure data for large biological molecules (proteins, DNA, RNA, viruses, macromolecular machines and their complexes). PDB holdings at the time of writing include >227 000 experimentally determined atomic-level 3D structures. These data are essential for research and education across fundamental biology, health, energy and biotechnology (1,3). RCSB PDB collaborates with its wwPDB partners (including Protein Data Bank in Europe or PDBe, Protein Data Bank Japan or PDBj, Protein Data Bank China or PDBc, Electron Microscopy Data Bank or EMDB and Biological Magnetic Resonance Data Bank or BMRB) to maintain a single PDB archive and ensure that this information remains freely and publicly available to the global community (4,5).

The wwPDB adheres to the principles of fairness-accuracy-confidentiality-transparency (FACT (6)) and Findability-Accessibility-Interoperability-Reusability (FAIR (7)), ensuring equitable sharing and responsible management of the 3D biostructure data. Information stored in the PDB is made available under the most permissive Creative Commons CC0 1.0 Universal License (https://creativecommons.org/licenses/by/4.0/), enabling researchers around the world to access and utilize the information at no charge and with no restrictions on its usage. Recognizing its long-standing commitment to high standards of data preservation, management and open access, the PDB is accredited by CoreTrustSeal, an international organization that certifies data repositories (https://amt.coretrustseal.org/certificates/). More recently, the PDB was recognized by the Global Biodata Coalition (https://globalbiodata.org) as a Global Core Biodata Resource, of ‘fundamental importance to the wider biological and life sciences community and the long-term preservation of biological data.’ PDB remains a vanguard in the open access movement.

RCSB PDB activities are organized around four user-facing services (8): Service 1, deposition and biocuration; Service 2, archive management and access; Service 3, data exploration; Service 4, training, outreach and education. Recently, the organization decided to expand from the existing four integrated, interdependent services to five with the introduction of the new Service 0, IT Infrastructure. This change was designed to streamline cyberinfrastructure operations supporting the entire organization.

As reported previously (3,9–11), our research-focused RCSB.org web portal now provides access to > 1M Computed Structure Models (CSMs) together with > 227 000 experimentally determined structures of biological macromolecules, obtained using macromolecular crystallography (MX), 3D electron microscopy (3DEM) or nuclear magnetic resonance spectroscopy. Development of the ModelCIF data format by the wwPDB partners in collaboration with community stakeholders (12) was key to accomplishing this integration effort, as it provided a way to seamlessly combine two kinds of 3D biostructure data, experimental and computational, while preserving the provenance and metadata that distinguish each type. Notably, the RCSB.org modular architecture dating from late 2020 (13) has proved to be highly extensible in its support of complex data and capable of accommodating a six-fold increase in data volume.

In this update prepared for publication in the 2025 *Nucleic Acids Research* Database Issue, we present new features and improvements developed and implemented since our last publication to our research-focused web portal RCSB.org (3).

## Results

RCSB.org functionality supports four overarching activities comprising 3D biological macromolecule data exploration: Searching, Browsing, Visualizing and Comparing. Updates presented herein relate to these four topics, with the most prominent new features being searching with user-provided atomic coordinates and newly-introduced tools for visualizing and understanding ‘Groups’ of related structures (both experimentally determined and computationally predicted). New integrations with external information resources are also presented. They augment the wealth of structural and functional annotations incorporated from 50 public-domain biodata repositories. Beyond user interface (UI) improvement features, we also briefly present tools that enable easier access to RCSB PDB Application Programming Interfaces (APIs).

### Structure and structure motif search for user-provided atomic coordinates

Our 3D structure similarity (14) and structure motif search (15) tools have been extended to support RCSB.org queries based on user-provided atomic coordinates, which can be uploaded in various standard formats. Previously, queries were restricted to structures available from RCSB.org (experimental PDB archive and/or select CSMs). Users can now work with atomic coordinates provided either by a publicly accessible URL (Uniform Resource Locator) or file upload. This new functionality provides a convenient and streamlined way to relate novel, previously uncharacterized 3D structures to the wealth of 3D biostructure information archived in the PDB, which includes expertly biocurated data and annotations from 50 trusted external resources, alongside >1 million CSMs coming from AlphaFold DB (16) and the ModelArchive (modelarchive.org) (17).

**Figure 1.**
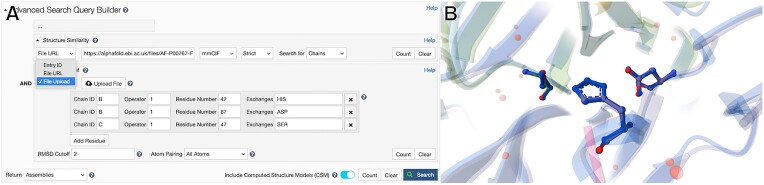
(**A**) Advanced Search Query Builder, showcasing support for searches defined by File URL and File Upload for Structure Similarity and Structure Motif searches. (**B**) Mol* 3D visualization example of a Structure Motif search match with the serine protease catalytic triad (search motif derived from PDB ID: 4cha (21)). Shown here is one of the returned matches, the AlphaFold CSM for bovine Chymotrypsinogen B (UniProt ID: P00767).

Search by URL or file upload is available in the Advanced Search Query Builder (Figure 1A) via the left-most dropdown menu under Structure Similarity or Structure Motif. The ‘File URL’ option can reference 3D structure data from external repositories (e.g. AlphaFold DB, the ModelArchive or the ESM Metagenomic Atlas (18)). Alternatively, the ‘File Upload’ option allows users to browse and select an atomic coordinate file locally stored on their computer, which is then automatically uploaded to the RCSB.org cyberinfrastructure and referenced using a bookmarkable and shareable URL. The Mol* 3D viewer (19) on RCSB.org (https://www.rcsb.org/3d-view) provides an alternative means of performing searches with user-provided atomic coordinates: after loading a user-provided structure via the Mol* interface, the structure gets automatically uploaded, and then custom Structure Motif searches can be performed (20). Regardless of the structure's origin, users can easily export the global alignment generated by the Structure Motif Search tool as a ZIP archive. To do this, use the ‘Export Models’ option in Mol*, which becomes available after selecting the ‘Align in 3D’ link on the search results page.

This file upload feature is powered by a dedicated RCSB PDB API service (user-upload.rcsb.org). Files can be uploaded in PDBx/mmCIF (22), BinaryCIF (23) or legacy PDB file formats, which are then parsed and converted with the aid of the BioJava library (24). Each successfully uploaded file is assigned a shareable URL with a randomly generated 32-character identifier to limit access. Uploaded files are accessible *via* these ‘non-guessable’ URLs for 90 days. Structure Similarity and Structure Motif searches can be defined using URLs as input. Generated queries can be bookmarked and shared while the uploaded file URL (s) remain valid. The User Upload API is also utilized within the pairwise structure alignment application (described below). At the time of writing, this User Upload API was supporting ∼20 000 external atomic coordinate file uploads monthly.

Overall, these enhancements to Structure Similarity and Structure Motif searches significantly expand the functionality and accessibility of RCSB.org cyberinfrastructure, providing researchers with powerful tools to explore, visualize, and analyze 3D biostructures with increased ease and flexibility.

### Pairwise structure alignment tool enhancements

**Figure 2.**
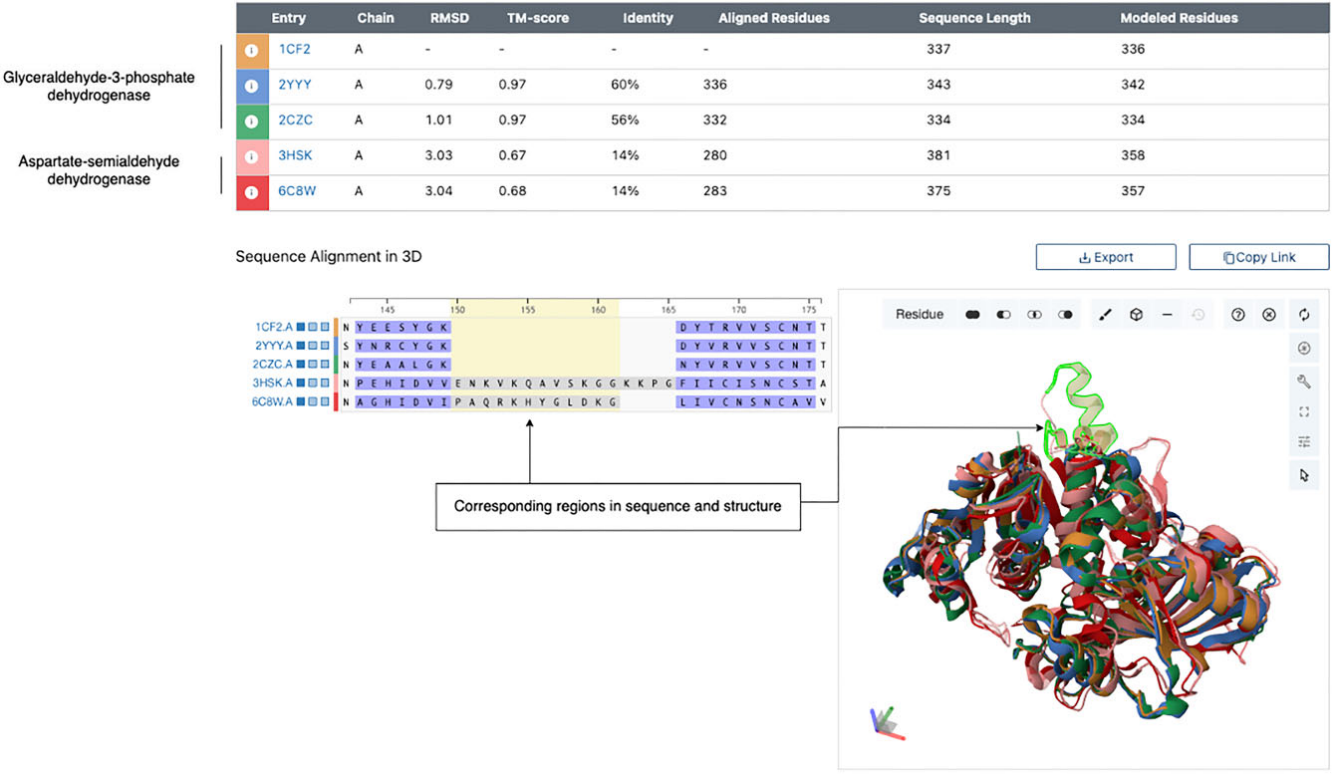
Alignment of structures to explore the NADP-binding sites of glyceraldehyde-3-phosphate dehydrogenase from *Methanothermus fervidus* (PDB ID: 1cf2 chain A, in orange, the reference structure (29)), *Methanocaldococcus jannaschii* DSM 2661 (PDB ID: 2yyy chain A (30), blue), *Pyrococcus horikoshii* OT3 (PDB ID: 2czc chain A (31), green), aspartate-semialdehyde dehydrogenase from *Candida albicans* (PDB ID: 3hsk chain A (32), salmon) and *Blastomyces gilchristii* SLH14081 (PDB ID: 6c8w chain A (33), red).

The RCSB PDB Pairwise Structure Alignment tool (Figure 2 (25)) was updated recently to provide more efficient options for constructing queries and additional interactive ways of exploring alignment results. We also introduced various improvements to streamline navigation through established alignment methods (*e.g*. TM-align (26) *versus* jCE-CP (27)). The tool now leverages the power of the RCSB PDB Sequence Annotations viewer (previously called 1D-3D viewer (28)) and Mol* (19) to facilitate bi-directional exploration between sequence alignments and structure superpositions. Key new features include synchronized highlighting of aligned sequences and superposed structures, the ability to show or hide aligned polymeric chains plus other polymers and non-polymers present in the PDB ID, and enhanced structure alignment visualization that distinguishes residues in spatially similar positions from those that are not.

To streamline access to the most recent predictions of protein 3D structures from AlphaFold DB (16) and ESM Metagenomic Atlas (18) for structure alignments, we introduced a feature that retrieves atomic coordinates directly via appropriate identifiers. Users can now input UniProt ID codes (34) to access AlphaFold DB and MGnify identifiers, access ESM Atlas and automatically locate and load the corresponding atomic coordinate files. Across RCSB.org, UniProt accession codes can be used to facilitate simultaneous selection of 3D biostructures of interest from among >227 000 experimentally determined PDB IDs and >1M incorporated CSMs (coming from both AlphaFold DB and ModelArchive). This feature facilitates comparison of ‘ground truth’ PDB structures with predicted atomic coordinates and enables fuller structural appreciation of many eukaryotic multi-domain proteins for which PDB coverage may not extend across the entire polypeptide chain length.

Other UI improvements were implemented to enhance navigation through available 3D structure alignment methods. Each option in the ‘Alignment Method’ dropdown now provides context-sensitive help that offers concise descriptions and guidance on usage. Available alignment methods are also grouped to clearly distinguish between rigid-body methods and methods designed to permit flexibility within proteins (e.g. FATCAT Flexible *versus* FATCAT Rigid (35)).

Importantly, RCSB.org users now can see the exact API request being issued to the RCSB PDB Alignment API service (alignment.rcsb.org) for calculating 3D structure alignments, after hitting the ‘Compare’ button on the Pairwise Structure Alignment page. The ‘Alignment API’ button opens a dedicated query editor page that provides a vehicle for further customizing API queries, enabling users with varying levels of technical expertise to utilize the Alignment API. Similar API buttons are available elsewhere across RCSB.org (described in the section entitled *API discoverability*).

### Exploring Groups of similar proteins

The PDB archive has captured the outcomes of a multitude of structure determination experiments over more than five decades. Given funding emphases and challenges of trying to determine structures of some biological macromolecules, the archival contents do not provide uniform representations of protein and nucleic acid structure shapes. Many PDB IDs are structurally similar if not nearly identical (e.g. PDB holdings include >1450 independently determined X-ray crystal structures of the SARS-CoV-2 main protease, the target of nirmatrelvir, the active ingredient of Paxlovid (36), beginning with PDB ID: 6lu7 (37)). To help users make sense of this wealth of information, RCSB.org supports exploration of ‘Groups’ of structures (10).

The RCSB.org Advanced Search interface and Search API both provide tools that manage cases in which searches return PDB IDs that are similar in 3D structure. Different approaches are supported, depending on whether the search returns *Structures* or *Polymer Entities*. When the search return type is set to ‘Structure’ (meaning PDB ID), the results can be organized based on the deposition Group identifier denoting collections of structures deposited simultaneously *via* GroupDep (38). When set to ‘Polymer Entities’, results can be grouped based on amino acid sequence similarity clusters (39) or collections of structures that share the same UniProt ID. Once search results are grouped, results can be presented as a list of Groups or as a list of representative members of each Group. The search configuration also offers different options for selecting Group representatives, including best experimental resolution (i.e. lowest number in Å for MX or 3DEM structures), largest total residue count, largest number of chains in the PDB ID, most extensive polypeptide chain coverage (when grouping by UniProt ID) or highest search relevance Score (as judged by our Search API system, based on the Elasticsearch relevance calculation). When RCSB.org Advanced Search presents results as Groups, each result item summarizes the Group, displaying relevant features such as Group name, the number of Group members satisfying the search conditions and the diversity of source organisms, domain families and Enzyme Classification (EC) numbers represented in the Group. Additionally, the title of the returned search result items also serves as a link to the Group Summary Page (GSP) associated with the grouping strategy.

Examining collections of proteins with shared similarities can uncover patterns in both sequence and 3D structure that may be missed when proteins are analyzed individually. RCSB.org provides a robust set of tools for analyzing and visualizing Groups of proteins. These Groups are defined using the same strategies implemented for ‘redundancy’ reduction in search results:

- Deposition Groups: Sets of structures that researchers deposited simultaneously using the GroupDep tool (38).
- UniProt Groups: Encompass all proteins in the PDB and/or CSMs incorporated into RCSB.org with amino acid sequences sharing the same UniProt ID.
- Sequence Identity Groups: Defined by calculating sequence identity clusters of protein sequences across structures in the PDB archive and/or CSMs incorporated into RCSB.org.

Group tools now available at RCSB.org were developed to enable comprehensive understanding of structural and functional annotations across multiple members of a Group. They facilitate visualization of Group member property distributions, multiple sequence alignments (MSAs) and 3D structure superpositions. Group tools are accessible from GSPs, which serve as the main hub for exploring all Group-related features, including Group MSA visualization and Group 3D structure superpositions. GSPs can be accessed from structure summary pages (SSPs) and Advanced Search results displayed as Groups. SSPs link each of their protein sequences (PDB IDs, which are listed under the Macromolecules section) to their corresponding UniProt and Sequence Identity GSPs. When Advanced Search results are presented as Groups, the listed result items link to their corresponding GSP based on the selected grouping strategy (Deposition, UniProt or Sequence Identity).

**Figure 3.**
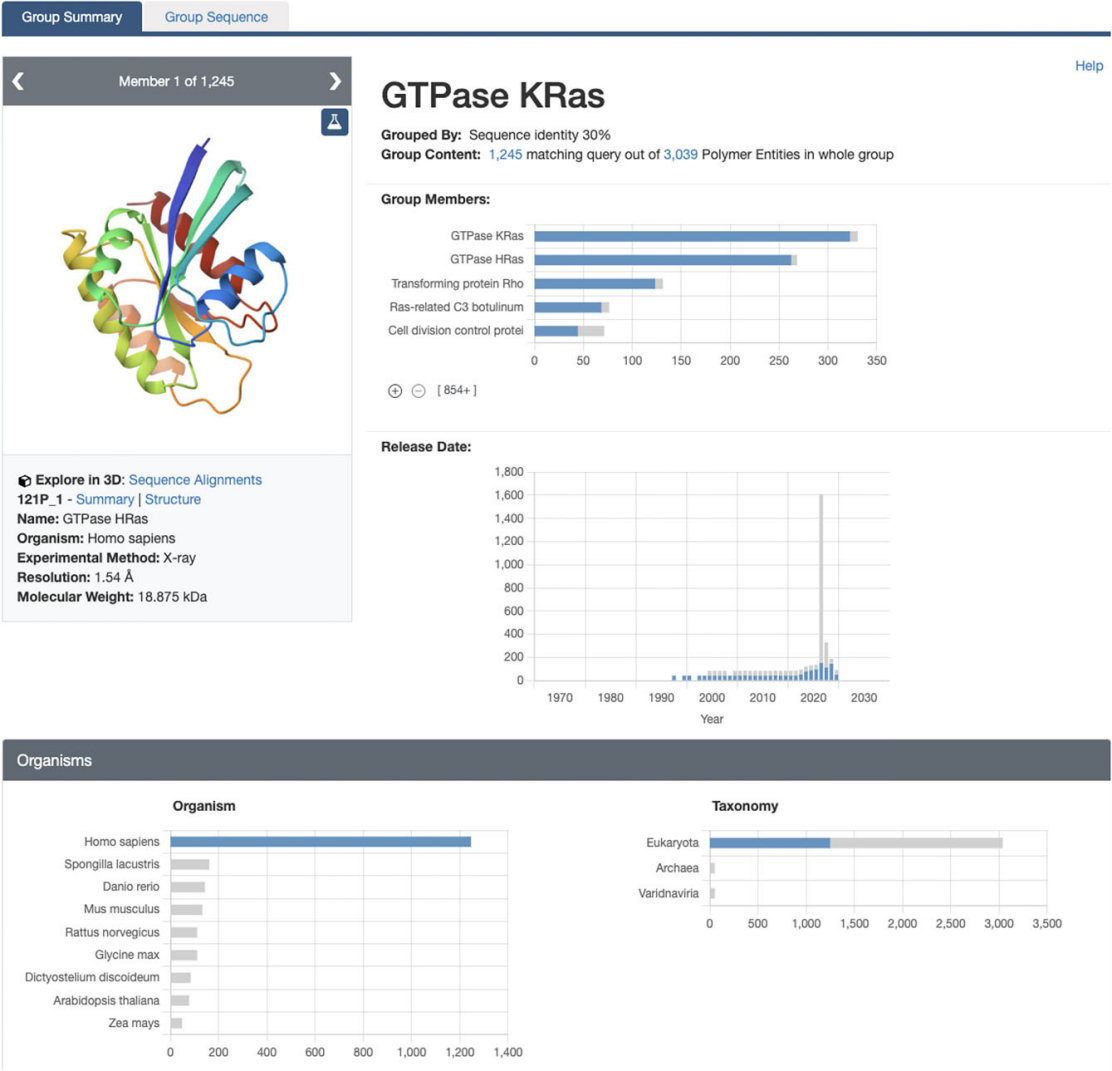
GSP for a 30% sequence identity cluster of Ras family proteins. Histograms display the distribution of organisms among the Group members, with the currently filtered *Homo sapiens* displayed in blue. Gray bars represent the distribution of Group members belonging to other organisms.

GSPs (Figure 3) serve as the primary entry point for exploring Groups and Group-related resources, such as Group Sequence Pages and Group Sequence Alignments in 3D. GSPs contain all the data and metadata related to the Group and its members, including both experimentally determined PDB structures and CSMs. The GSP layout is similar to SSPs, featuring a page header, a carousel of images of each Group member (top-left), and various sections with key information about the Group member properties. Each header showcases the Group title, the methodology used for grouping members, the total count of elements within the Group and, when accessed from the Advanced Search, the number of Group elements matching the search query. The ‘carousel’ component enables exploration of individual Group members. Each slide in the carousel displays specific details about a Group member, including name, organism and key experimental information such as resolution and molecular weight. Group member properties are represented as histograms that plot the distribution of specific properties of the Group members (e.g. resolution, release date or source organism). These histograms are organized into different sections displaying distribution of related features. For example, the ‘Protein Domains’ section shows the distribution of protein domains within the Group members based on various protein domain classification databases, including CATH (40), SCOP/SCOPe (41,42), ECOD (43) and Pfam (44).

When GSPs are accessed from Advanced Search results, histograms display distributions using two different colors: blue represents the distribution of the Group members that matched the search query, while Group members that do not match the search are displayed in gray (Figure 3). In addition, histograms allow exploration of subsets of a distribution. Clicking on a histogram bar will update all plots to display the distribution for the Group members related to the selected property. For example, if the X-ray Crystallography bar is clicked in the Experimental Method histogram, all histogram plots will be updated to display the distribution of the Group members determined using X-ray Crystallography. A floating navigation menu ‘Query History’ allows going back to the previous unfiltered view.

Group Sequence Pages are available for UniProt- and sequence identity-based Groups. These pages use the Sequence Annotation Viewer (45) to provide detailed sequence information for Group members, divided into three main sections. The Sequence Alignment tab displays a graphical representation of the MSA of Group members, helping users visualize how sequences align across the Group and highlighting conserved and variable regions. The Structural Features tab shows the distribution of structural properties, such as secondary structure (e.g. $\alpha$-helices and $\beta$-sheets) and protein domains by using a color gradient to represent the frequency of occurrence at a certain position. The Binding Sites section displays the distribution of protein-ligand binding sites. The content displayed on Group Sequence Pages is interconnected with information from GSPs. When RCSB.org users select a specific property to inspect a subset of the initial Group, the Group Sequence Page updates to display relevant data for that subset, allowing for more targeted analyses and visualization.

**Figure 4.**
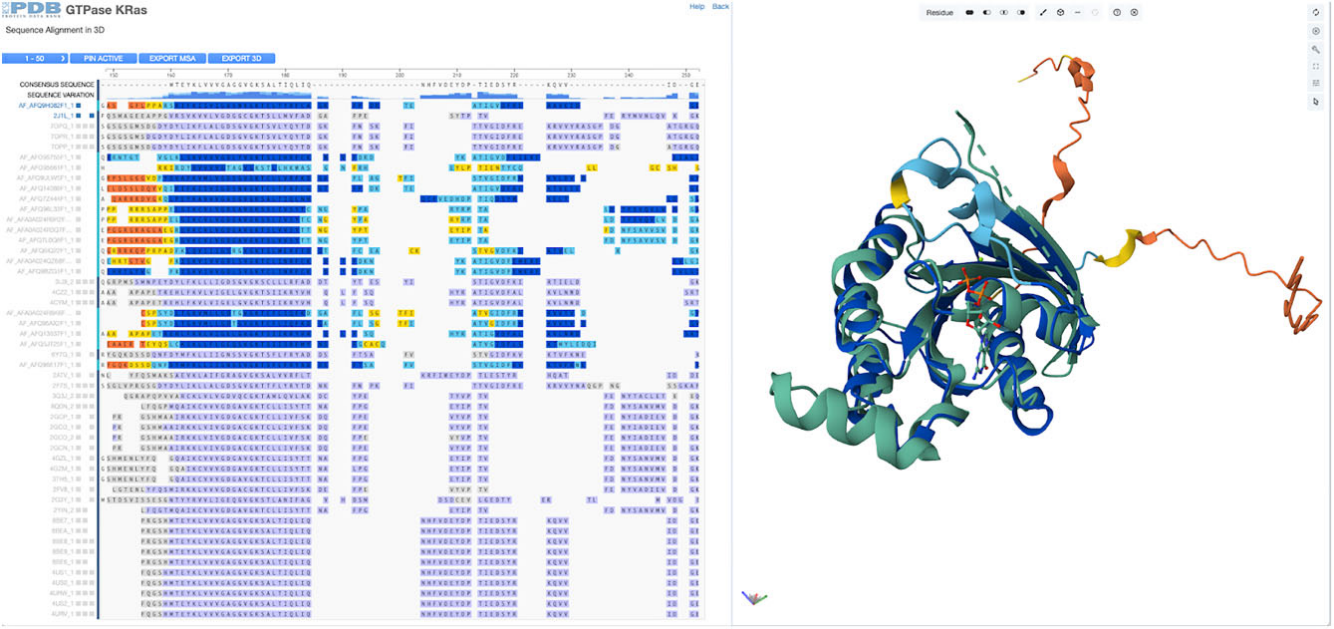
Group Sequence Alignments in 3D for Ras family proteins clustered at 30% amino acid sequence identity. On the left side, an MSA of the group members is displayed, including experimental structures and CSMs. The right panel shows the 3D structure of selected proteins from the alignment panel in Mol*.

The Sequence Alignments in 3D tool is designed to integrate sequence and structural data for Group members (Figure 4). This tool is also available for both UniProt- and sequence identity-based Groups and is accessible from the GSP carousel and the Group Sequence Pages by clicking the ‘Sequence Alignments in 3D’ link. This tool utilizes the RCSB PDB 1D3D (45) to integrate the Sequence Annotation Viewer and the Mol* 3D viewer, presenting the MSA of Group members with their 3D structures. MSA visualization mirrors the information provided on GSPs, with the added functionality of clicking on track titles to load Group member 3D structures into the Mol* 3D viewer. Newly loaded structures are superimposed on the reference structure (first loaded), facilitating comparisons of Group members in 3D. The UI for this tool is identical to that used for the Pairwise Structure Alignment tool (see *Pairwise Structure Alignment tool enhancements* above).

### Integration of data from new external resources

In addition to providing access to 3D structure data and related metadata, RCSB.org integrates information from numerous open-access bioinformatics resources and makes these data available for users to map onto 3D structures and visualize them. Depending on the type of annotation, integrated annotations may be presented for specific structures (entry-level annotations) or at the level of the biopolymers (entity- or instance-level annotations) present in the structure. These annotations can be used to learn about biomolecular structural attributes (e.g. CATH domains, membrane binding regions), functional properties (e.g. EC numbers, anatomical therapeutic classification of drugs) and locations of special significance (e.g. sites of post-translational modification, commonly observed variants of the polymer sequence) (3). Newly integrated annotations enable users to explore antibiotic resistance and mechanisms of enzyme catalysis across the PDB archive. Including these annotations along with other criteria to construct complex queries can help identify structures relevant to diverse user research interests.

The Comprehensive Antibiotic Resistance Database (CARD (46)) is a highly curated database of antibiotics and antibiotic resistance genes, their protein products, and phenotypic information, organized by the CARD Antibiotic Resistance Ontology (‘ARO’). Polymer sequences of proteins present in PDB IDs that perfectly or closely match (>95% amino acid sequence identity and >80% polypeptide chain coverage) the antibiotic resistance proteins referenced in CARD are identified and linked to specific annotations, such as the matched protein gene name, ARO identifier, description, resistant antibiotic drug classes and the biochemical mechanism of resistance. When sequences of the protein in the PDB and the reference Antimicrobial Resistance Gene have <95% sequence identity, only gene family annotations are included at the Annotations page. Links at RCSB.org to the CARD database provide access to additional details about these annotations. Identifying related antibiotic resistance proteins can provide valuable insights into biochemical/biological function.

**Figure 5.**
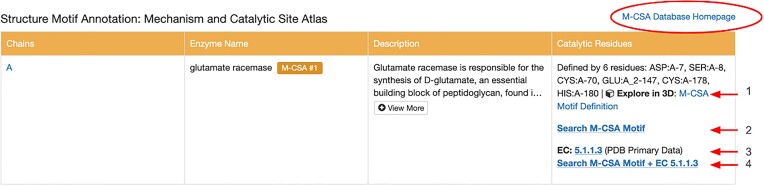
Tabular representation of M-CSA annotations for PDB ID: 1b73 (48). Links allow the user to (1) visualize the catalytic residues in Mol*; (2) launch a structure motif search based on the M-CSA motif definition; (3) launch a search for other structures in the PDB with the same EC number; (4) and launch a search with both the structure motif for similar arrangements of catalytic residues and PDB IDs or CSMs bearing the same EC number.

The Mechanism and Catalytic Site Atlas (M-CSA) resource is a database of enzyme reaction mechanisms (47). It also provides annotations concerning active site and catalytic residues plus enzyme reaction cofactor requirements. Each one of the ∼1000 entries stored in this external resource is linked to at least one experimentally-determined structure archived in the PDB. Linked PDB IDs are annotated with details from the M-CSA, which can in turn be used to query other structures (experimentally-determined from PDB or CSMs from AlphaFold DB or the ModelArchive) that have active-site 3D structure motifs similar to the enzyme of interest. Structure motif searches launched in this manner can help an RCSB.org user identify and learn about other PDB structures with comparable active site architecture. For example, in PDB ID: 1b73 (48), which includes an M-CSA annotation, the Catalytic Residues column can be used to search for other PDB structures or CSMs that have the same catalytic site residues and/or the same EC number, using options shown in Figure 5.

**Figure 6.**
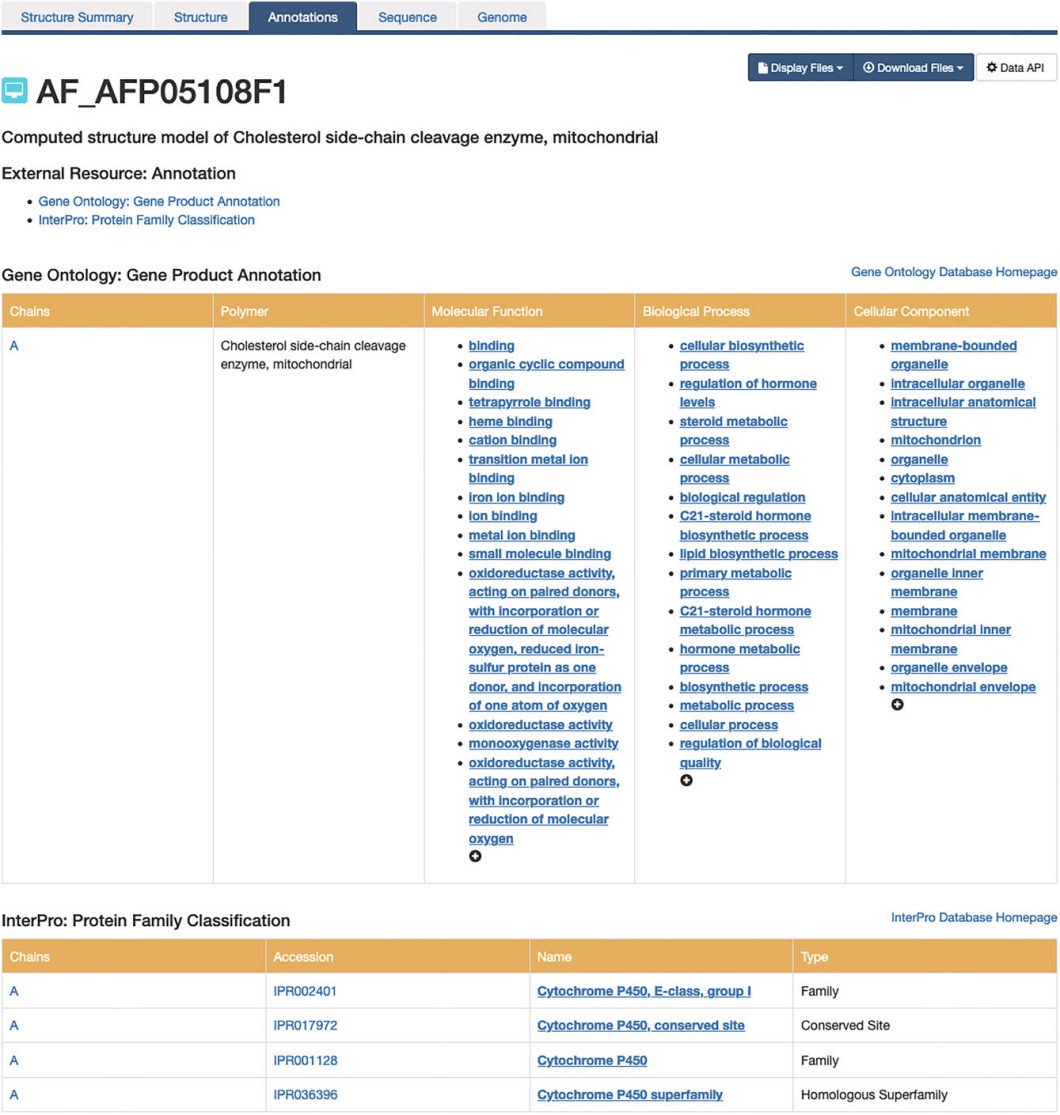
Annotations available for a CSM of cholesterol side chain cleavage enzyme, mitochondrial (AlphaFold: AFP05108F1).

Recently, annotations of 3D structures available from RCSB.org were extended to CSMs. Thus polymer sequence-level annotations derived from the UniProt are mapped to CSM structures available from RCSB.org and made accessible from the SSP Annotations tab. Some of these annotations include Gene Ontology (49), InterPro (50) and Pharos (51), and can provide new perspectives about the CSMs being examined (e.g. Figure 6).

### Support for extended chemical component identifiers

Several improvements for exploring PDB chemical components (small molecule chemicals) and their specific instances within individual PDB IDs have been added recently. The first, transparent to end-users but vitally important for software providers, concerned extension of PDB Chemical Component IDs from three to five characters. Three-character identifiers were predicted to be fully exhausted in late 2023, thus the wwPDB implemented necessary changes in PDB structure deposition, validation, and biocuration processes to extend the length of the identifier to five characters. With >60 million combinations, Chemical Component IDs are effectively future-proof. Our RCSB.org research-focused web portal and all APIs that underpin features therein were made fully compliant with the five-character ID in late 2023 when the first such five-character ID was publicly released. As of June 2024, the PDB had issued 416 such Chemical Component IDs, represented at the time within 479 distinct PDB IDs.

Importantly, due to limitations in the legacy PDB format, PDB structures containing the new five-character Chemical Component IDs will only be provided in PDBx/mmCIF format. Transitioning to the PDBx/mmCIF format is highly encouraged. wwPDB resources (https://mmcif.wwpdb.org/docs/user-guide/guide.html) are available to assist data consumers, software providers, etc. in moving away from the legacy PDB format.

The Ligand Summary Page, the main landing page for exploration of Chemical Components at RCSB.org, now utilizes Mol* to unify the 3D visualization experience across our research-focused web portal. Users can also toggle views between ideal (from the Chemical Component Dictionary) and model atomic coordinates.

A further user experience improvement to ligand exploration was recently introduced at the SSPs ‘Small Molecules’ section, wherein the ‘3D Interactions’ buttons are now drop-down menus that allow selection of specific ligand instances and two different ways of visualizing the atomic coordinates: with experimental electron density (now presented in a wireframe style by default), or atomic coordinates-only.

### Incorporation of additional computed structure models

In previous publications (3,9–11), we described expansion of structural coverage at RCSB.org through the incorporation and parallel delivery of ∼1 000 000 CSMs alongside a collection of the then-available ∼195 000 experimentally-determined, rigorously validated, and expertly-biocurated PDB structures. The CSMs included in this release encompassed AlphaFold2 (52) predictions of entire proteomes for model organisms, common human pathogens, crop plants and nearly all of Swiss-Prot (coming from AlphaFold DB (16), https://alphafold.ebi.ac.uk/), plus a set of predicted yeast protein binary complexes generated using a combination of RoseTTAFold and AlphaFold2 (53,54) coming from the ModelArchive, thereby broadening structural coverage at RCSB.org substantially.

This expansion of coverage is critically important for RCSB.org users interested in model organisms with modest PDB coverage. Sole reliance on experimentally-determined PDB structures of *Arabidopsis thaliana* proteins, effectively limits 3D structural coverage of that proteome to ∼4%. With incorporation of *A. thaliana* CSMs from AlphaFold DB, RCSB.org now provides plant molecular and cellular biologists with access to 3D structure information across the entire proteome. Similar coverage enhancements are now benefitting researchers focused on studying proteins from *C. elegans, D. melanogaster, M. musculus, S. pombe*, etc.

Following this initial CSM release, additional datasets (encompassing tens of thousands of new computationally predicted protein structures) have been incorporated into RCSB.org as part of our commitment to serving as a one-stop shop for the study of 3D structures of biological macromolecules. These datasets (all coming from the ModelArchive) include predicted protein structures from *Spongilla lacustris* (freshwater sponge) (55), African swine fever virus (56) and *Sphagnum divinum* (peat moss (57)), as well as a set of heterodimeric proteins from the cancer interactome in humans (58). Collectively, these additions have contributed ∼68 000 CSMs, bringing the total number of CSMs available on RCSB.org to ∼1 068 000. Importantly, owing to the development and adoption of the ModelCIF data standard (the extension to the PDBx/mmCIF data standard developed for computationally predicted protein structures) (12), every one of these CSMs could be fully integrated at RCSB.org and made interoperable with the same set of powerful tools for search, browsing, analysis and visualization available for the extant collection of ∼223 000 experimentally determined PDB structures.

### API discoverability

SSPs and Ligand Summary Pages now contain a new button ‘Data API’ (located at the top-right), which opens the GraphQL query to RCSB PDB Data APIs in its query editor. This new feature improves discoverability of APIs for users who want to learn how certain data can be extracted using APIs and to understand how information is structured within RCSB PDB data schemas. Beyond the main SSP tab, similar buttons are offered in other commonly accessed tabs: Annotations, Experiment, Ligands and Versions. Analogously, the search result page offers a ‘Search API’ button on top-right to open (in the Search API query editor) the search query actually used to obtain the search results displayed in the current view. A common cogwheel icon styling across all these API buttons provides a visual cue to users that serves to identify the functionality.

Another recent development that offers API usability improvements at RCSB.org is our release of the py-rcsbsearchapi Python library (https://github.com/rcsb/py-rcsbsearchapi), a Python interface to the RCSB PDB Search API. Queries against the API can be constructed conveniently using either an operator interface or a fluent syntax. With access to this library, users can easily incorporate queries to the Search API in their own software or scripts.

### Training opportunities

RCSB PDB hosts webinars, virtual and in-person crash courses, and virtual office hours to support users in their research and training across disciplines. A recent event demonstrated how to use RCSB.org features to navigate CSMs in the context of experimentally determined PDB structures all in 3D (‘A Deep Dive into Computed Structure Model Exploration at RCSB.org’). Other events included an introduction to PDB structure quality metrics, including those presented in wwPDB Validation Report graphical sliders (‘Understanding PDB Validation: Which experimental structures should I rely on?’); ‘Visualize Biomolecular Structures with Mol*: From Atoms to Movies’; and ‘Python Scripting for Molecular Docking’. Webinars are recorded and published on PDB-101 (https://pdb101.rcsb.org/) in the ‘Train’ section. PDB-101 hosts training, outreach and education resources developed by the RCSB PDB that are focused on structural biology and related topics (59). It aims to build confidence in current and future users to promote more effective utilization of RCSB.org tools and 3D biostructure data writ large. Materials are developed for users at various skill levels, targeting graduate students, postdoctoral fellows and established researchers in subject areas ranging from data deposition to data exploration.

Other PDB-101 training materials include a highly popular *Guide to Understanding PDB Data*, which supports RCSB.org users who do not have strong backgrounds in structural biology or data science. Available articles span from a ‘Beginner's Guide to PDBx/mmCIF’ (the format that drives the PDB archive and RCSB.org) to a focus on biological assemblies. New articles are published online as major features are added to RCSB PDB, such as an ‘Introduction to RCSB PDB APIs’ and ‘Computed Structure Models’ (a.k.a. CSMs).

Future training events are announced at RCSB.org, PDB-101, and on social media; would-be attendees can also subscribe to our training events newsletter at https://pdb101.rcsb.org/train/training-events or info@rcsb.org to receive additional notices.

## Data Availability

No new data were generated or analyzed in support of this research. Resources described are freely available at RCSB.org.
